# Cell Surface Markers in Colorectal Cancer Prognosis

**DOI:** 10.3390/ijms12010078

**Published:** 2010-12-28

**Authors:** Larissa Belov, Jerry Zhou, Richard I. Christopherson

**Affiliations:** School of Molecular Bioscience, University of Sydney, Sydney, NSW 2006, Australia; E-Mails: jzho7551@mail.usyd.edu.au (J.Z.); R.Christopherson@usyd.edu.au (R.I.C.)

**Keywords:** colorectal cancer, biomarkers, proteomics, prognostic

## Abstract

The classification of colorectal cancers (CRC) is currently based largely on histologically determined tumour characteristics, such as differentiation status and tumour stage, *i.e.*, depth of tumour invasion, involvement of regional lymph nodes and the occurrence of metastatic spread to other organs. These are the conventional prognostic factors for patient survival and often determine the requirement for adjuvant therapy after surgical resection of the primary tumour. However, patients with the same CRC stage can have very different disease-related outcomes. For some, surgical removal of early-stage tumours leads to full recovery, while for others, disease recurrence and metastasis may occur regardless of adjuvant therapy. It is therefore important to understand the molecular processes that lead to disease progression and metastasis and to find more reliable prognostic markers and novel targets for therapy. This review focuses on cell surface proteins that correlate with tumour progression, metastasis and patient outcome, and discusses some of the challenges in finding prognostic protein markers in CRC.

## 1. Introduction

Colorectal cancer (CRC) is a cancer of epithelial origin, localized to the large intestine and rectum. It is one of the most common cancers and will occur at some stage in approximately 5% of the population of the western world. The conventional prognostic factors for patient survival are histologic tumour grade (differentiation) and tumour stage (TNM, tumors/nodes/metastases, stages I–IV) [1,2], which is based on depth of tumour invasion, involvement of regional lymph nodes and metastatic spread to other organs [3]. If metastasis has occurred, patient 5-year survival after surgery falls dramatically from 90% to less than 10% [4]. It is therefore important to increase our understanding of the molecular changes leading to development, spread and metastasis of CRC and to identify potentially prognostic and predictive markers for the disease.

Despite the discovery of a range of intra- and extra-cellular protein biomarkers for CRC and continuing efforts to discover potentially prognostic and predictive markers using various approaches [5–12], the translation of this increasing volume of differential proteomic data into patient care remains a major challenge. This is partly due to the heterogeneous nature of CRC [13,14] and the complexity of processes involved in its development and spread, but also to challenges presented in detecting and characterising glycoproteins and low abundance proteins [15,16] in complex mixtures. In addition, validation of candidate proteins as prognostic markers is time-consuming and expensive, and recent critical reviews of differentially expressed proteins from comparative proteomic studies have suggested that some reported proteins may be associated with general cellular stress responses, rather than being disease-specific biomarkers [17,18]. The development of a reliable assay for determining prognosis and guiding post-surgical therapy therefore remains elusive.

The metastatic potential of CRC may be already encoded in the primary tumour [19], and molecular staging using a classifier based on 43 genes has identified patient prognosis more accurately than the traditional clinical staging, particularly for intermediate stage II and III patients [20]. It may be possible to identify disease signatures based on protein expression profiles in primary CRC tissues that reflect potential for disease progression and metastasis, and subsequently use these signatures to predict patient recovery and survival after surgical removal of the primary CRC tissue. With this goal in mind, we have developed a 122-antibody microarray (DotScan™ CRC microarray; Medsaic Pty Ltd, NSW, Australia) for immunophenotyping live cells from fresh disaggregated CRC tissues [21,22]. Microarrays prepared as 10 nl antibody dots on nitrocellulose-coated microscope slides (FAST; Grace Bio-labs, Bend, OR, USA) bind cells with corresponding surface molecules, producing a binding pattern that reflects the surface immunophenotype of the mixed cell population. Bound cells are then fixed to the microarray with formalin, and specific sub-populations are profiled by multiplexing with mixtures of soluble fluorescently-labelled antibodies. Hierarchical clustering of binding patterns for these sub-populations of cells yields patient clusters that may correlate with disease stage, tumour invasiveness, differentiation, drug susceptibility or patient outcome. This antibody-based multiple marker approach has already been validated for the classification of a range of common human leukaemias and lymphomas [23].

The evolution of CRC from an adenomatous polyp to metastatic disease is dependent not only on progressive accumulation of genetic and epigenetic abnormalities [24], but also on the complex interactions of different sub-populations of cells within the tumour microenvironment (cancer cells, normal stromal cells, infiltrating leukocytes and the soluble factors they produce) [25]. The importance of cell surface molecules for cell-cell adhesion and communication between cancer cells and non-malignant cells has been recently reviewed [26,27], and the significance of signalling pathways involved in CRC progression as a result of these interactions has also been described [28–32]. Since many drugs used in cancer therapy target cell membrane proteins [33], this review seeks to summarise current knowledge on cell surface molecules that may serve as therapeutic targets, have prognostic significance or provide important information about the invasive, metastatic and proliferative potential of cancers of the large intestine and rectum. Antibodies to some of these proteins have been incorporated into the current DotScan™ CRC antibody microarray; others could be added in the future. Readers are also referred to several previous reviews on markers of metastasis and prognosis in CRC [34,35].

## 2. Tumour Stroma

Recently, the importance of the tumour microenvironment for the development, promotion and spread of cancer has become clear [36], and the potential for using tumour stroma-derived biomarkers in prognostication and therapy of cancer patients has been realised [37]. The stroma consists of the non-malignant cells of the tumour—fibroblasts, infiltrating leukocytes and cells of the blood vessels. However, despite experimental evidence for the importance of tumour stroma for cancer development and progression, clinical applications for stromal biomarkers remain limited. Down-regulation of TGF-beta receptor 2 (TGF-β-R2) in tumour-associated stroma correlates with a worse prognosis [38], while expression of platelet-derived growth factor receptor (PDGFR) on stromal cells correlates directly with metastatic potential and advanced-stage disease in CRC [39,40].

Stromal CXCR4 (CD184) and CXCL12 expression is associated with distant recurrence and poor prognosis in rectal cancer after chemotherapy [41]. Patients whose colon tumours have high levels of stromal fibroblast activation protein (FAP) are more likely to have aggressive disease progression and potential development of metastases or recurrence [42]. Urokinase receptor (uPAR; CD87) expression in stromal fibroblasts has been implicated in the occurrence of haematogenous metastasis (dissemination via the blood) of CRC [43]. The presence of high levels of stroma and down-regulation of Deleted in Pancreatic Cancer 4 (SMAD4) predict worse survival for early-stage CRC patients [44]. Expression of CD10 (a cell surface metalloprotease) on stromal cells adjacent to cancer cells within the area of invasive growth is significantly higher in invasive CRC than in non-invasive CRC or adenomas [45]. The stromal expression of CD10 also appears to be significantly associated with abnormal accumulation of nuclear p53 in tumour cells and a larger tumour size, suggesting that CD10 expression may contribute to tumour invasion and possibly facilitate metastasis [45].

Thrombospondin-1 (TSP-1), mainly localised in fibroblasts of the CRC stroma [46], has been considered as an important negative-regulator of tumour angiogenesis. TSP-1 expression in primary CRC tumours has been correlated inversely with metastatic potential and prognosis [47–51]. In CRC liver metastases, however, TSP-1 expression has been linked to significantly poorer survival, suggesting that TSP-1 in liver metastases may have an alternative mode of action, facilitating tumour invasion rather than acting as an anti-angiogenic growth factor [51].

## 3. Inflammation and Infiltrating Leukocytes

Inflammatory reactions in tumours can have a positive influence on survival [52,53] or alternatively can be associated with development of metastases and disease progression [54–56]. The role of tumour-associated macrophages (TAM) in tumourigenesis is complex because TAMs can either prevent or promote tumour development. While Forssell *et al.* [52] showed that a dense TAM infiltration at the tumour front positively influenced prognosis in colon cancer, strong pro-tumourigenic effects of TAMs in CRC have also been reported [54–57]. It has been suggested that the balance between pro- and anti-tumourigenic properties of TAMs may depend on their interaction with cancer cells, other stromal cells, and the tumour microenvironment [54] or be influenced by the degree of cell-cell contact [52].

The presence of high numbers of mature CD208+ infiltrating dendritic cells (DC) in the tumour epithelium has been associated with shorter overall patient survival, while patients with high numbers of CD1a+ DCs in the advancing margin of the tumour appear to have a shorter disease-free survival [58]. These findings are controversial, however, as a strong correlation has also been found between the presence of DCs together with high density T-cells and no detectable metastases [59] and better patient survival [60].

The presence of large numbers of tumour infiltrating lymphocytes (TILs) at the invasive tumour front has generally been associated with a good prognosis for CRC patients [61–64]. Patients with high levels of CD3+, CD8+, CD25+, CD45RO+ or CD95L(CD154)+ TILs appear to have a better clinical course [65–69] than those with low levels, particularly in early disease stages [70]. The importance of the intra-tumoural location, immunophenotype and density of these infiltrates has been demonstrated [71–73].

It has been shown that CD3+ TILs located in the tumour stroma are not as strong predictors of prognosis as T-cells located within cancer cell nests (clusters), consistent with the requirement for direct contact between target and effector cells [71–74]. In addition, although the presence of high density CD3+ TILS in primary tumours has been associated with good prognosis in patients with lymph node-negative CRC, there was no significant correlation between high density CD3+ TILs and absence of post-surgical metastases in patients with lymph node-positive CRC [75].

Patients with T-cells showing low CD4+/CD8+ ratios have a better clinical course than those with high ratios [59,76]. The infiltration of cancer cell nests by CD8+ T-cells has been associated with better survival [67], supporting the importance of activated cytotoxic CD8+ T-cells in the anti-tumour response to CRC [77,78].

The better overall survival of CRC patients with high infiltrates of CD3+ (total) and CD8+ (cytotoxic) T-cells within cancer cell nests, was recently confirmed [70]. Interestingly, however, this correlation was not significant for the rectal cancer patient sub-group when it was analysed separately from the colon cancer group. Furthermore, in patients whose tumours were positive for microsatellite instability (MSI+), a hallmark of DNA mismatch repair (MMR) deficiency [79], no association was found between high levels of activated cytotoxic T-cells and a favourable prognosis. Although these findings are in agreement with those of several other groups, they are in conflict with a number of studies in which high infiltrates of CD8+ TILs correlated with good prognosis regardless of MSI-status [70].

Marked infiltration of CD8+ and CD57+ natural killer (NK) or NK-like T-cells in the advancing tumour margin has also been associated with longer disease-free survival, and this was most evident in MSI+ tumours [80]. Metastatic spread of CRC is associated with decreased NK-cell activity [81], but it remains speculative whether decreased NK-cell activity precedes the development of metastases and may therefore be prognostic.

Koch *et al.* [82] reported strong and significant enrichment for CD4+ T helper cells in CRC compared to corresponding normal mucosa, *i.e.*, decreased proportions of CD8+ cells in total T-cell populations of CRC. However, a significantly higher proportion of the CD8+ TILs in CRC expressed markers of activation (CD69+) and cytotoxic activity (CD107a+) compared with normal mucosa. Significantly, the proportion of activated CD8+ TILs decreased with tumour stage [82].

If a tumour exhibits a high T-cell infiltrate, the prognosis for patients with strong tumour expression of major histocompatibility antigen class I (MHC 1) is better than for those who have weak MHC 1 expression [74], because the immune response mounted against the tumour cells is weaker in these patients.

TILs recovered from primary tumours showing no sign of metastasis differ immunophenotypically from those showing early signs of tumour metastasis (presence of vascular emboli, lymphatic invasion and perineural infiltration). Using flow cytometry, Pages *et al.* [68] found that TILs from tumours with no sign of metastasis showed higher expression of markers of cell adhesion (CD62L, CCR7, CD103, CD49d and CXCR3), activation (HLA-DR, CD98, CD80, CD86 and CD134) and differentiation (CD45RO, CD45RA, CD27, CD28, CCR7 and CD127). Primary CRC tumours without early signs of metastatic invasion such as vascular emboli (*i.e.*, infiltration of cancer tissue into blood vessels) presented with significant increases in T-cell sub-populations from early memory (CD45RO+CCR7- CD28+CD27+) to effector memory CD8+ T-cells (CD45RO+CCR7-CD28+CD27-). Using immunohistochemistry-based tissue microarray analysis of 415 tumours, they confirmed that a high density of memory T-cells (CD3+CD45RO+) in the primary tumour correlated with the absence of early signs of metastatic invasion, confirming previous findings [83]. In a comparison of patients with high T-cell infiltration, primary tumours from patients with metastases had significantly decreased densities of CD8+ T-cells and effector memory T-cells (CD27-CD45RA-) than patients without metastases [59].

The surface antigen expression of total TILs differs from that of T-cells in peripheral blood, with markedly lower expression of CD29, CD49d and CD49f, and slightly higher expression of CD49a and CD49b [84]. CD11a and CD18 are reduced on CD8+, but not CD4+, sub-populations [84]. TILs also differ from lymphocytes within normal colon interstitium by lacking CD28, CD56, CD98 and CD154 [85] and showing altered expression of CD3, CD29, LFA-1 (CD11a/CD18) and LFA-3 (CD58) [86].

The function and relative importance of different CD4+ sub-populations in CRC is still only partially understood. Although CD4+ TILs are observed in CRC [59,80,87], the density of this sub*Int.* population has shown little prognostic value in CRC to date, and is not significantly different in primary tumours of patients with metastatic or non-metastatic disease [59]. A high density of CD4+ TILs in CRC liver metastases has correlated with poorer patient outcome [63].

Recent attention has focussed on CD4+CD25+ regulatory T-cells (Treg), which are characterised by expression of nuclear transcription factor forkhead box P3 (FoxP3). The CD3+/FOXP3+ cell ratio [88] and the CD8+/FOXP3+ cell ratio [89] are both predictive markers for disease-free survival time and overall survival time in patients with CRC, supporting the down-modulatory role of Treg in the presence of protective cytotoxic T-cells. Controversially, the presence of a high density of FoxP3+ Treg in CRC has also been associated with improved survival in stage II and stage III CRC patients [66,83] and in relapsed CRC patients undergoing chemotherapy or chemoimmunotherapy [90]. Although this result is unexpected and counterintuitive, it has been suggested that the increase in Tregs in tumour tissue may represent a feedback response to a pre-existing cancer cell-directed immune response [90]. Frey *et al.* [91] found that high FOXP3(+) Treg density was associated with early tumour stage and independently predicted improved disease-specific survival in MMR-proficient CRCs, but not in MMR-deficient CRCs, though high Treg density correlated with absence of lymph node involvement and vascular invasion in the latter. They suggested that the role of Treg may differ according to the clinical stage and the MMR status of CRC.

We have prepared live cell suspensions from enzymically disaggregated primary tumour tissue from CRC patients and corresponding normal mucosa. DotScan™ CRC antibody microarrays and fluorescence multiplexing were used to profile the cells, and the CD3+ subset showed differential expression of HLA-DR, TCRα/β, CD49d, CD52, CD49e, CD5, CD95, CD28, CD38 and CD71 in descending order of difference [21].

## 4. Cancer Cells

### 4.1. Tetraspanins and Potential Tetraspanin-Associated Proteins

Tetraspanins are small transmembrane proteins, such as CD9, CD37, CD53, CD63, CD81, CD82, CD151 and tetraspanin 8, that are involved in a multitude of biological processes, including cell-cell adhesion, metastasis suppression and tumour progression [92–96]. Clinical studies have established a link between tetraspanin levels and prognosis and/or metastasis in cancer. The tetraspanins CD82 and CD9 mostly suppress tumour progression; their expression is often reduced in late-stage human tumours and their down-regulation during CRC progression is associated with poor prognosis [97]. CD151 and tetraspanin 8 support tumour progression; increased CD151 is an indicator of poor prognosis.

Tetraspanins interact with each other and other proteins that are required for their function, to form complexes in tetraspanin-enriched membrane domains (TEM) [95]. This has been referred to as the ‘tetraspanin web’ [93,94]. The most prominent non-tetraspanin partners are integrins, e.g., β1 (CD29), α3 (CD49c), α4 (CD49d), α6 (CD49f) and β4 (CD104); matrix metalloproteinases (MMP), e.g., MT1- MMP (MMP14); members of the Ig superfamily, e.g., ICAM-1 (CD54) and VCAM-1 (CD106); as well as CD26, CD44, EpCAM (CD326), E-cadherin (CD324), beta-catenin (β-catenin) and ADAM10. Some of these molecules have been implicated in tumour metastasis and poor patient prognosis.

Down-regulation of E-cadherin, the prime mediator of epithelial cell-cell adhesion [98], correlates with a strong invasive potential and poor prognosis in CRC [99–101]. High CRC cell staining for β-catenin, a binding partner of E-cadherin [102], is independently associated with better patient survival [103]. In contrast, membranous expression of β-catenin, together with low E-cadherin, is associated with CRC tumour progression, and may be an independent prognostic factor for poor patient outcome [104].

Absence of CD49c correlates with reduced overall and disease-free survival compared to patients with CD49c+ CRC [105]. CD29 is commonly down-regulated in CRC compared to normal intestinal mucosa [22,106,107], and this has been correlated with invasion and metastasis [108]. However, there is little evidence for correlation between CD29 levels and patient outcome, possibly because up-regulation of CD29 has also been correlated with lymph node metastasis and depth of invasion in CRC [106]. The association of CD104 with members of the tetraspanin web also appears to support tumour cell motility and liver metastasis [109].

CD44 over-expression in CRC is generally considered to be an unfavourable prognostic factor for overall survival, though this may depend on the isoform. CD44 variant 6 (CD44v6) expression correlates with poor prognosis [110–112]. However, loss of CD44 significantly correlates with poor survival when associated with a corresponding loss in E-cadherin, especially in Stage II CRC, suggesting that CD44 and E-cadherin may have an inter-dependent role in suppressing invasion and metastasis [113]. The worst prognosis was associated with low E-cadherin coupled with high MT1-MMP (MMP14) [113], itself associated with tumour invasiveness in CRC [114–116].

Low numbers of CD54-expressing tumour cells may be associated with a shorter disease-free survival [117]. The prognosis of patients with CD54-negative tumours is significantly poorer than those with CD54-positive tumours, suggesting that low CD54 expression is closely associated with metastasis and may be a useful indicator of prognosis [118]. Infiltration by TILs has been more frequently observed in the CD54-positive than in CD54 negative tumours.

### 4.2. Epithelial-Specific Cell Adhesion Activation Molecule (EpCAM, TROP-1, CD326)

EpCAM is a pan-epithelial differentiation antigen which promotes cell adhesion [119]. In normal epithelial tissues, increased EpCAM expression is associated with enhanced proliferation and a lower differentiation grade of epithelial cells. In the proliferative phase, EpCAM expression is associated with epithelial tissue remodelling. After cell proliferation, EpCAM expression declines and cellular differentiation commences [120].

The regulation of EpCAM in tumour invasion and metastasis has previously been reviewed [121–125]. In carcinogenesis, EpCAM over-expression is present during the early stages, but dramatically decreases during malignant transformation and progression. Over-expression of EpCAM reportedly correlates with biological aggressiveness and poor prognosis in CRC [126], possibly by promoting tumour immune evasion by strongly favouring T-helper cell type 2 (Th2) development that promotes growth of EpCAM-expressing tumors [127]. However, a reduction in surface expression of EpCAM has also been associated with aggressive cancers and poor prognosis in CRC [120]. The hypothesis that loss of EpCAM expression may be involved in cancer metastasis is further supported by the finding that EpCAM protein expression on circulating tumour cells (CTC) is reduced compared with primary and metastatic tumours [128].

Using a monoclonal antibody to detect an extra-cellular epitope of EpCAM in rectal tumours, Gosens *et al.* [120] found that decreased membranous EpCAM staining in the “budding” cells at the invasive tumour front was always accompanied by a focally infiltrating growth pattern, correlated with a significantly higher risk of local recurrence and indicated an elevated risk of distant recurrence. Importantly, loss of membranous EpCAM coincided with increasing cytoplasmic staining at the tumour front (detected by polyclonal antibody), suggesting altered cellular localisation due to abnormal post-translational processing of EpCAM rather than reduced expression of this antigen. An alternative view is that regulated intra-membrane proteolysis results in the shedding of EpCAM’s ectodomain and the nuclear translocation of its intracellular domain, resulting in mitogenic signal transduction [129].

This decline in membranous EpCAM expression at the tumour front was associated with nuclear β-catenin localization [120], which is associated with reduced cell–cell adhesion and increased migratory potential [130]. It did not correlate with disease stage, but did correlate with lower tumour differentiation grade, and was strongly prognostic [120]. It was predominantly observed in budding tumour cells or clusters. Tumour budding is a term used to describe the appearance of foci of de-differentiated cancer cells at the invasive tumour front of differentiated adenocarcinomas, *i.e.*, epithelial-to-mesenchymal transition (EMT) [131–133], and is frequently linked to high-grade tumours, lymph node positivity, vascular and lymphatic invasion and local and distant metastases [134].

Decreased membranous EpCAM staining throughout the tumour rather than just at the invasive margin also correlates with increased risk of local recurrence, but is not indicative of distant recurrence [120]. Interestingly, increased EpCAM expression has been linked to CRC progression when it is associated with claudin-7 and recruited into TEM where it complexes with CD44v6 and tetraspanin 8 [135,136]. This complex formation may facilitate metastasis by preventing EpCAM-mediated cell-cell adhesion and promoting migration, proliferation, apoptosis resistance and tumourigenicity.

### 4.3. Mucins, Carbohydrates, Lectins

Cancer cells, especially adenocarcinomas, tend to over-express mucins or express aberrant forms of mucins, the function and significance of which have been reviewed [137–139]. In CRC, high mucin content has been associated with more aggressive phenotype and poorer prognosis [140]. CD227 (MUC1), a membrane-associated mucin that displays altered *O*-glycosylation patterns in carcinomas, presents as an independent prognostic factor of CRC [141,142]. MUC1-Tn epitope becomes exposed during malignant transformation to adenocarcinoma and is associated with poor clinical outcome [143]. CD175s (sialosyl-Tn) can modulate a malignant phenotype inducing a more aggressive cell behaviour, and is associated with poor clinical outcome [144]. MUC2 has been associated with less aggressive tumour phenotypes and improved survival in some studies [61,145], although reverse findings have also been reported [146].

CD15s (Sialyl-Lewis^x^, sLe^x^) has an important role in defining the invasion and metastasis of human CRC; high level expression has been significantly correlated with metachronous metastases after surgery and poor prognosis [147–150]. The incidence of CD15s positive primary tumours was correlated with the depth of tumour invasion, lymph node metastasis, lymphatic invasion, and the disease stage [150]; but Baldus *et al*. [141] did not find CD15s to be an independent prognostic factor of CRC.

Sialyl-Lewis^a^ (sLe^a^; CA 19-9), a mucin epitope whose levels in CRC correlate with overall survival and disease free survival, may serve as an indicator of metastatic potential [141,151]. It is believed that tumour metastasis and cancer cell arrest in the vasculature is facilitated through the tethering of circulating cancer cells to vascular endothelium through the interaction of molecules such as CD15s, CA 19-9 and CD44 variants on the tumour cell surface and selectins (CD62-P, -L and –E) expressed on endothelial cells, neutrophils, monocytes, NK cells or platelets [152–155].

Galectin-3, a lectin, is involved in neoplastic transformation and tumour progression and has demonstrated significant prognostic value, particularly in stages I and II CRC [141,156,157]. Low levels of galectin-3 expression in the tumour epithelium are associated with significantly better prognoses than those characterized by high levels [158]. Galectin-3 up-regulates MUC-2 expression [159], and a functional link has been demonstrated between galectin-3, MUC-2 and metastasis in animal models [160,161]. However, these findings contradict other reports of an inverse relationship between MUC2 expression and tumour aggressiveness [61,162].

### 4.4. Carcinoembryonic Antigen (CEA) Family

The best known member of this group is carcinoembryonic antigen (CEA; CD66e), which is up-regulated relative to normal intestinal mucosa in 94% of CRC samples [163]. However, the significance of CEA over-expression in CRC as a prognostic marker remains controversial. Despite several reports supporting a possible correlation between CEA expression in CRC and tumour spread or patient survival [164–166], no definitive correlation has been established [163,167] and CEA has found little utility as a prognostic marker. However, the expression of CEA by disseminated tumour cells isolated from intra-peritoneal lavage was found to strongly correlate with advanced tumour stages and an extremely poor prognosis [168], consistent with the suggestion that CEA may enhance metastasis by functioning as an attachment factor for disseminated CRC cells [169].

CD66a (CEACAM1) and CD66c (CEACAM6) are also over-expressed in 58% and 55% of patients, respectively [163]. While no prognostic significance has been associated with CD66a, the over-expression of CD66c was found to be significantly associated with worse prognosis in a group of patients who received peri-operative FU-based chemotherapy [163]. CD66c has also demonstrated a role in tumour cell migration, invasion and adhesion, and formation of distant metastases [170]. CEA-related cell adhesion molecule 7 (CEACAM-7) is down-regulated in rectal cancers relative to normal mucosa and independently predicts recurrence-free survival for stage II rectal cancers [171].

### 4.5. Annexins

Annexins A1, A2, A4 and A11 are expressed at higher levels in CRC than in normal colon, and have been implicated in tumour development and progression [172]. Over-expression of Annexins A4 and A11 [172], Annexin A2 (Annexin II) [173] and Annexin A5 [174] may serve as markers of poor prognosis in patients with CRC.

### 4.6. Claudins

Claudin-1 and Claudin-4, transmembrane proteins important for tight junction formation in normal mucosal epithelial cells [175], are both up-regulated in CRC compared to normal mucosa, and this up-regulation is associated with down-regulation of E-cadherin and significant disorganisation of tight junction fibrils [176]. The over-expression of claudin-1 and -4 occurs not only in the plasma membrane, but also in the cytoplasm [176], and its function in CRC is not yet defined. However, loss of claudin-1 expression is a strong predictor of disease recurrence and poor patient survival in stage II CRC [177], while a decrease in claudin-4 at the invasive front in CRC has been associated with cancer invasion and metastasis [178].

### 4.7. Chemokine Receptors

Expression of the chemokine receptors CXCR3 [179,180] and CXCR4 (CD184) [181,182] has been associated with metastasis and poorer patient prognosis in CRC. Stroma-derived chemokines such as stromal cell derived factor-1 (a ligand of CXCR-4), may induce growth and metastatic progression of tumours by modulating proliferation, survival and homing, through chemokine receptors expressed on cancer initiating cells [183]. In contrast, chemokine receptor 5 (CCR5) expression has been associated with non-metastatic CRC and increased CD8^+^ T-cell infiltration [184].

### 4.8. Growth Factor Receptors

Epidermal growth factor receptor (EGFR) plays an important role in the biology of CRC [185] and is being used as a target for therapeutic antibodies in CRC patients with unmutated *KRAS* genes, but not in those with *KRAS* gene mutations which cause resistance [186,187]. However, despite reports indicating its value as a prognostic marker [188,189], its potential has not been realised [190]. Although patients with EGFR expression have a higher risk for disease recurrence compared to those with EGFR negative tumours, there appears to be no relationship between EGFR expression and overall survival [191,192].

A significant correlation has been demonstrated between insulin growth factor receptor (IGFR) expression levels (membrane plus cytoplasmic) and lymph node metastasis in CRC patients, especially when analysed in combination with vascular endothelial growth factor (VEGF) and VEGF-C [193].

Although cytoplasmic over-expression of human epidermal growth factor receptor 2 (CD340, HER-2/neu, ErbB2) is reported to be an independent prognostic indicator in CRC [194], membranous expression of this protein shows no prognostic significance [195]. Membranous expression of receptor tyrosine-protein kinase ErbB4, however, has recently been reported as an independent prognostic factor for recurrence in CRC patients after radical surgery [196].

Hepatocyte growth factor receptor (Met) expression correlates with poor overall survival in advanced stages of CRC [197]. In stage I and II patients, however, membranous Met alone does not appear to be a significant predictor of patient outcome, although the membranous to cytoplasmic Met ratio correlates with survival [198].

### 4.9. Death Receptors (DR) and Ligands

The roles of death receptors and their ligands in cancer onset, progression and therapy have been reviewed [199]. Low expression of death receptor 4 (DR4; TRAILR1; CD261) has been linked to poor prognosis in CRC [200], consistent with a tumour suppressor role for DR4 and its ligand, tumour necrosis factor related apoptosis-inducing ligand (TRAIL). However, TRAIL has shown no prognostic significance in several studies [201,202] and has even correlated with worse overall survival in stage II and III CRC patients [203]. Interestingly, high DR4 expression has also been associated with worse disease-free and overall survival in stage III adjuvant-treated CRC patients [202]. These findings are consistent with the proposed existence of an alternative TRAIL signalling pathway in TRAIL-resistant cells, which leads to cancer promotion rather than inhibition [204,205]. The prognostic significance of the up- or down- regulation of these molecules may therefore vary in different patient sub-groups.

Loss of CD95 (Fas) in tumour cells is related to adverse prognosis and maybe an independent prognostic factor in CRC [206]. Fas-ligand (CD178; CD95L; FasL) expression, however, correlates with lymph node involvement and distant metastases in CRC [207]. It has been suggested that the up-regulation of FasL in CRC cells can induce apoptosis of Fas-expressing T lymphocytes in the tumour [208]. Low Fas and high FasL maybe a useful indicator of lymph node metastases and poor prognosis in CRC [209].

### 4.10. Major Histocompatibility Complex (MHC)

The major histocompatibility complex (MHC) codes for human leukocyte antigens Class I (HLA-A, -B, -C) and Class II (HLA-DR, -DQ, -DP). Better prognosis correlates with the down-regulation of Class I antigens [210,211] and strong HLA-DR expression [212,213] on CRC cells.

### 4.11. Other Protein Markers in CRC

The A33 antigen is found in 95% of primary and metastatic CRC and also in the normal intestinal mucosa [214]. A33 is a promising target for immunotherapy [215,216] and radioimmunotherapy [217,218] of CRC, but A33 levels have not been associated with prognosis.

CD10 expression in CRC correlates with liver metastasis [219–221] and with the development and progression of CRC [162], but its prognostic significance in CRC has not been confirmed [222]. CD13 (aminopeptidase N; APN) is involved in cell motility and angiogenesis, and CD13 expression may be a useful indicator of a poor prognosis for node-positive patients with colon cancer [223]. Membrane complement resistance factors CD55 and CD59 have been reported as markers of tumour aggression, and have been associated with reduced survival in CRC patients [224,225]. CD55 acts as a cellular ligand for CD97; strong CD97 expression by tumour cells at the invasive front has been correlated with a higher clinical stage and increased lymph vessel invasion compared to cases with uniform CD97 staining throughout the tumour [226].

Membranous expression of the cell adhesion molecule CD166 (ALCAM), a member of the immunoglobulin superfamily, has been correlated with significantly shorter survival time in CRC patients [227]. Cell adhesion molecule CD171 (L1) expression is also associated with tumour progression and poor prognosis in CRC patients [99,228], and has been used, in combination with E-cadherin and membranous β-catenin, to identify stage II patients who may benefit from adjuvant chemotherapy [104].

Deleted in colorectal cancer protein (DCC) has been proposed as a putative tumour suppressor, and loss of DCC expression has been associated with poor prognosis and risk of metastasis [229]. Expression of DCC is a strong positive predictive factor for survival in CRC [230,231].

Several novel membrane proteins have recently been identified in CRC. ATP-binding cassette sub-family G member 5 (ABCG5) is a membrane protein involved in efflux transport of cholesterol. ABCG5 expression in tumour buds has recently been associated with significantly poorer prognosis in node-negative CRC patients [134]. ATP11A is an integral membrane ATPase, probably involved with the transport of ions such as calcium across membranes. Patients with high ATP11A expression have shown poorer disease-free survival compared to those with low expression, indicating that an increase in ATP11A expression may be an independent predictor of metastasis in CRC patients [232].

## 5. Colorectal Cancer Stem Cells (CSC)

Cancer stem cells have been identified in several human malignancies, including CRC, and are believed to drive tumour initiation, progression and metastasis [233–238]. Tumour buds, discussed above, appear to have many features of CSC, and the possibility that a sub-population of tumour buds may represent malignant stem cells is an open question requiring further investigation [133].

The presence of a sub-population of CD26+ CSC in primary CRC tumours was found to predict distant metastases on follow-up [239]. These cells were associated with enhanced invasiveness and chemoresistance. High EpCAM expression was also associated with oncogenic potential in CSC [122], and CD166 (ALCAM) was found to be differentially expressed on EpCAM^high^/CD44^+^ CSC [240]. Other CSC markers, CD133 and CD24, correlated with invasiveness and differentiation in CRC, while CD44 expression was related to tumour burden [241]. No correlation was found between the expression of these three markers and patient survival [241], but others have shown that the combined evaluation of CD133, CD166 and CD44 could be used to identify high and low survival among stage I and II CRC patients [242]. Although CD133+ cells have demonstrated CRC initiating potential [243], CD133 expression may not be restricted to cancer-initiating cells [244]. In addition, highly metastatic CD133- sub-populations of cancer-initiating cells have been identified in a human CRC xenograft model, suggesting that CD133+ tumour cells might give rise to a more aggressive CD133- subset during the metastatic transition [244].

CD200 (OX2) is a membrane glycoprotein that induces an immuno-regulatory signal through its receptor (CD200R), leading to the suppression of T-cell-mediated immune responses. CD200 has been associated with tumour progression and poor prognosis in several cancers [245,246] and is over-expressed in CRC with markers for CSC [247], suggesting that tumour-initiating CSC may evade immune system detection through CD200-induced suppression of T-cell-mediated immune responses.

## 6. CRC Translational Proteomics Research

A number of proteomic studies on CRC tissues have identified differentially expressed proteins from whole tissue samples of CRC and paired normal mucosa [8,16,248–252]. In other studies, enrichment for specific sub-populations of cells has been performed using techniques such as laser micro-dissection [253,254], fluorescence activated c*ell sorting* (*FACS*) [240] or antibody-conjugated magnetic beads [240,255]. Interpretation of results is complicated by the fact that different methods have been employed for sample preparation, antigen detection and protein quantification. For antibody-based detection methods, the choice of monoclonal antibody is important, as differences in staining may reflect changes in epitope structure due to mutations, alternative splicing or post translational modifications, rather than up- or down-regulation of protein expression. In addition, some changes in protein activities during disease progression may correlate with altered sub-cellular localisation of proteins [120]. Thus the use of different methods of protein quantification may lead to conflicting results.

Methods used for membrane proteomics in cancer biomarker discovery have been previously reviewed [26]. Due to the hydrophobicity of membrane glycoproteins and their relatively low levels compared to soluble intracellular proteins [256], the identification and validation of novel differentially expressed surface markers in CRC has been limited. Membrane fractions from patient CRCs have been analysed using two-dimensional fluorescence difference gel electrophoresis (2D-DIGE) saturation labelling followed by MALDI-TOF-TOF mass spectrometry, with identification of 23 proteins, many classified as membrane or membrane-associated [5]. Others have been identified from membrane CRC fractions by iTRAQ [248]. Although approaches such as 2D-DIGE and mass spectrometry have led to the discovery of a number of differentially expressed proteins in CRC [5–8,10–12,257,258], the prognostic significance of these molecules and their contribution to cancer growth and metastasis has not always been easy to establish. Hence very few of these proteins have found clinical utility either as prognostic markers or therapeutic targets.

Proteomic methodologies and their application in CRC research have been reviewed, and the limitations of traditional approaches for cancer biomarker marker discovery and future challenges have been discussed [259–261]. It has been suggested that multiple markers will be required to enable reliable sub-classification of cancers [262–264]. The use of immunohistochemistry for analysis of CRC tissue sections for multiple potentially prognostic/predictive markers [141,265,266] has the advantage of revealing antigen location; however the method is relatively low through-put, expensive, labour-intensive, and quantification is difficult. The use of tissue microarrays enables analysis of a large number of samples simultaneously, but the number of antigens that can be detected in a single assay is limited [267].

**Figure 1:**
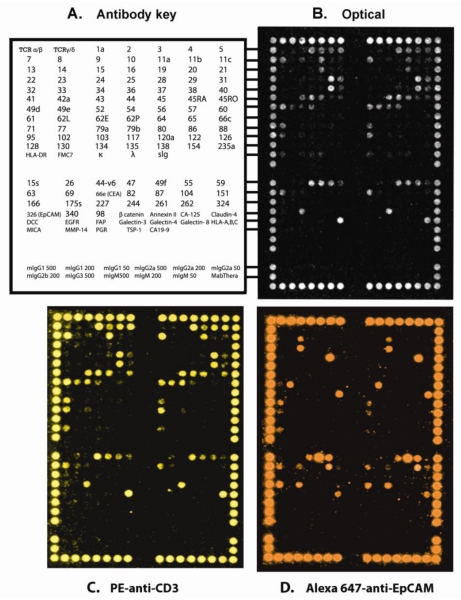
Binding patterns of a disaggregated stage II, poorly differentiated, CRC sample on a DotScan™ CRC antibody microarray. (**A**) Antibody locations for duplicate arrays. Numbers refer to CD antigens; other abbreviations are TCR, T-cell receptor; κ, λ, immunoglobulin light chains; slg, surface immunoglobulin; DCC, deleted in colorectal cancer protein; EGFR, epidermal growth factor receptor; FAP, fibroblast activation protein; HLA-A,B,C and HLA-DR, human leukocyte antigens A,B,C and DR, respectively; MICA, MHC class I chain-related protein A; MMP-14, matrix metallopeptidase 14; PIGR, polymeric immunoglobulin receptor; TSP-1, thrombospondin-1; Mabthera, humanised anti-CD20. Alignment dots around microarray consist of a mixture of CD44 and CD29 antibodies. (**B**) Optical scan; (**C**) and (**D**) fluorescence multiplexing using PE-anti-CD3 and Alexa 647-EpCAM, respectively.

The DotScan™ CRC antibody microarray allows rapid immunophenotyping for 122 surface antigens on a population of disaggregated live tumour cells in suspension, requiring 4 × 10^6^ cells per assay. Density of cell binding per antibody dot depends on the proportion of positive cells in the population, level of antigen expression and affinity of the antibody. Figure 1 shows optical and fluorescent binding profiles for a CRC sample from a stage II patient. The optical binding pattern reflects the immunophenotype of the mixed cell population, while the CD3-PE and EpCAM-Alexa Fluor 647 staining detect T-cells and cancer/epithelial cells, respectively. The limit of detection is approximately 1 cell in 10 for the optical scans, while the sensitivity of fluorescent scans is four times higher. We have demonstrated sub-types of CRC based on hierarchical clustering of microarray patterns [22] that may provide prognostic information when data are retrospectively analysed with patient outcomes. This study is ongoing. With the addition of antibodies against newly discovered markers of CRC, this method could be used to correlate cell binding densities with disease stage, invasion, metastasis and patient outcome, and also for finding multiple-marker signatures allowing sub-classification of CRC.

Cell surface proteins are logical candidates as biomarkers and therapeutic targets; they enable a cell population to interact with other cells and soluble factors in the tumour microenvironment and reflect important genomic, epigenetic, transcriptional and translational changes during disease progression. As an alternative to the use of viable cells for analysis with the DotScan CRC antibody microarray, tumour cell-derived membrane fragments, microvesicles or microparticles could be used, as demonstrated with plasma-derived microparticles from patients with cardiovascular disease [268]. This alternative approach may facilitate the detection of potential prognostic biomarkers shed from cells located at the invasive front. Samples of the invasive front are required for routine pathology analysis and are not readily available for research purposes.

Zlobec and Lugli [133] have described the invasive front of CRC as a dynamic interface between pro- and anti-tumour factors. Tumour budding at the invasive front promotes progression and dissemination of CRC cells into the vasculature and lymph nodes, while infiltrating immune cells, particularly cytotoxic T-cells, mount an immune response. The CD8+ lymphocyte/tumour-budding index appears to be an independent prognostic factor in CRC [269]. E-cadherin expression is lower in tumour budding sites than in the centre of the tumour [270], possibly contributing to reduced cell-cell adhesion at the tumour front. It has been suggested that a comparison of the pro- and anti-tumour factors at the invasive front could contribute to development of a prognostic score for patients with CRC [133], with proteomic analysis providing new prognostic markers for CRC.

An alternative proteomic approach is the analysis of patient blood plasma or other biological fluids for CRC biomarkers [271], which include cancer-associated soluble factors, as well as tumour-derived microvesicles [272–275]. Prefractionation of biological fluids is required to detect low abundance proteins [276]. Lectin glycoarrays have been used to profile plasma for CRC biomarkers, with the identification of significant glycosylation changes associated with tumour progression [277–281].

In summary, the search continues for a reliable method of predicting metastatic spread and patient outcome after surgical resection of primary CRC. There is a general consensus that patterns of multiple biomarkers will be required; innovative approaches are needed to translate accumulating data into a useful method for sub-classifying CRC patients for disease stage and those likely to benefit from available treatments. Further progress in cell surface protein discovery will supplement new discoveries provided by other innovative technologies, such as metabolomics [282,283], study of oncogenic pathways [30,31], anti-sense therapeutic strategies [9,284], and combined proteomics/genomics strategies [285].

## Abbreviations

CEA: carcinoembryonic antigen
CD44v6: CD44 variant 6
CCR: chemokine receptor
CRC: colorectal cancer
CSC: colorectal cancer stem cell
CXCR: chemokine CXC motif receptor
DC: dendritic cells
2D-DIGE: two-dimensional difference gel electrophoresis
DR: death receptor
EpCAM: epithelial-specific cell adhesion activation molecule
FoxP3: forkhead box P3
MHC: major histocompatibility complex
HLA: human leukocyte antigen
Met: hepatocyte growth factor receptor
MMP: matrix metalloproteinase
MMR: DNA mismatch repair
MSI: microsatellite instability
NK: natural killer
TAM: tumour-associated macrophage
TEM: tetraspanin-enriched membrane domains
TIL: tumour infiltrating lymphocyte
Treg: regulatory T-cell
